# The Buffalo Academy of Medicine in 1908-091Presidential address delivered at the annual meeting held June 8, 1909.

**Published:** 1909-09

**Authors:** E. A. Bowerman

**Affiliations:** Buffalo, N. Y.


### BUFFALO MEDICAL JOURNAL.

VOL. LXV. SEPTEMBER, 1909. NO. 2

### ORIGINAL COMMUNICATIONS.

### The Buffalo Academy of Medicine in 1908-09.[1]

By E. A. BOWERMAN, M. D., Buffalo, N. Y.

IT becomes my pleasant duty at this time to review the work of one of the busiest and most successful years in the history of the Academy. At the close of last year the affairs of the Academy were left in a most satisfactory condition. The interest of the members had been stimulated by an excellent program, the attendance at the meetings had been increased and a feeling of good-fellowship permeated our membership. The way had been well prepared for still greater progress during the present year. Our membership at that time was 257. The interest in our organisation is shown by the fact that the membership has not suffered decrease from failure of members to pay their annual dues. We have lost but two members by resignation; one by death. During the year there have been received 85 resident and non-resident fellows, making a total membership of 339 at the close of the year. The interest has so increased that our meetings have nearly doubled in average attendance over former years.

It was with much hesitation and misgiving that I assumed the duties of presiding officer a year ago, an office that had been held by our most distinguished members, many of them men of national and international reputations. A most loyal membership has rendered my duties easy and pleasant in the extreme. The spirit of good-fellowship and enthusiasm for the Academy, I believe, has never been equalled. The standing of our body has assumed the position it deserves in the profession of Buffalo and surrounding towns, that of the most active, progressive and representative medical body in Western New York.

The reports of the several sections and of the secretary and treasurer are so flattering and so indicative of progress and accomplishment that, lest you think the ego most dominant, I wish to place the credit where it belongs—with a most united, en-

1. Presidential address delivered at the annual meeting held June 8, 1909.

thusiastic and energetic body of associate officers by whom it has been my good fortune to have been surrounded.

Enthusiasm, interest and energy on the part of all the officers in their respective positions have been radiated and have engendered a like spirit in the membership body. During the summer vacation when the work of preparing a program for the year was first inaugurated, it was taken up by the section officers with unprecedented activity. No time, energy, or expense was spared by them to give us the best, most instructive and entertaining program which could be procured. At the expense of their professional duties, trips were taken to the larger medical centers and by personal solicitation some of the leading medical and surgical authorities of the country were induced to appear at our meetings. By personal effort they have gained the interest and coöperation of the members who have shown their appreciation by regular attendance at our meetings and, I trust, to their own advantage.

The council, comprising the official body, has been free from drones, all have worked energetically and harmoniously for the good of the Academy. I wish personally to express my thanks to each and every member of the council who has so loyally assisted in carrying out the work of the year.

Perhaps one of the most important steps has been the reception to membership of men of recognised schools of medicine other than the regular or allopathic. This movement was started last year when a few of the homeopathic school were received. It has not been, however, until the present year that they were made to understand that the Academy sincerely desired the membership of every doctor in Buffalo and vicinity, who was a graduate of a school recognised by our profession and whose professional and moral qualifications met our standard. Our homeopathic brethren have accepted the invitation in the spirit in which it was extended and a majority of their number practising in Buffalo have become members. They have attended the meetings, presented papers, taken part in discussions and shown a lively interest in the affairs of the Academy, which has proven of mutual advantage to all concerned. I feel, if nothing else has been accomplished during the year, we have succeeded in a large measure in promoting a feeling of good-fellowship and in fostering a unanimity of interest in the profession at large. The mistaken feeling that the Academy belongs to certain cliques and factions has been dispelled. An attempt has been made to make all feel that however dissimilar their views, all were entitled to and would receive a cordial hearing in open debate.

I wish at this time to extend a cordial welcome to all of the homeopathic and eclectic professions who have joined our number, and to congratulate the profession upon their good sense in abolishing the bigotry and narrow mindedness which has heretofore held the various schools aloof. Such a move can not but make for a more united and intelligent profession.

The work of the commissions and special committees is of great interest. Some of these were appointed by my predecessors and by vote of the Academy continued during the present year. Others were created at the last annual meeting. Noteworthy among these in point of energy and successful work has been the commission to further the needs of the city for a contagious hospital. The chairman, Dr. Ullman, and his associates have devoted an amount of time and energy to this work which none but themselves know and none but those who have followed closely their efforts can appreciate. The initiative in this work was taken by the Academy and the coöperation of the Medical Society of the County of Erie followed. By their combined efforts various civil societies, clubs, and labor organisations were interested. Popular sentiment showing the great need of this hospital was aroused. The Common Council was memorialised and public hearings were largely attended as a result of their efforts. The city authorities were convinced, in the face of existing epidemics, that such an institution is a necessity.

At the request of the Common Council and Mayor Adam a bill was passed by the legislature authorising a bond issue to meet the cost of such an institution. A civil commission has been appointed to work out the details of the hospital. The selection of a site is under way. Progress in this enterprise has been rapid and continuous and it is only a matter of time before this project, originated in the Academy one year ago, will be an established fact looking toward the advancement of our city. The work of this committee has met many obstacles and difficulties. However, their unflagging energy has assured results which will work for the betterment of our city for all time. The thanks not only of the Academy but of the municipality is due these gentlemen.

No less energetic or untiring have been the efforts of the committee on permanent home for our Academy. This committee was created three years ago and its present members have served continuously during the entire period. The hope was entertained that we might secure a suitable meeting place of which our members could be proud. None but Dr. Jewett knows the amount of effort he has expended upon this project. He has been most ably seconded by the other members of the committee, who have attended faithfully its meetings and labored it its behalf. The need of such a meeting place which would be comfortable and attractive to our members is recognised. Its needs have many times been demonstrated during the past year when this room has been crowded. We have on many occasions suffered shame at being obliged to bring our distinguished visitors to this unattractive, poorly ventilated, subterranean den. After much careful deliberation this committee selected a site on which they obtained an option. Provisional plans for a building were prepared by Messrs. Esenwein & Johnson. Let me pause to express our thanks and appreciation to these gentlemen for this work which was gratuitous. These plans were submitted to our members and subscriptions requested. In spite of hard times and financial depressions, than whom none are more affected than our profession, many liberal subscriptions were received. More than half of the requisite amount was pledged by eighty of our members.

No systematic canvass of the entire membership was made by the committee as this was impossible. Many of our members whose pledges were not received I am sure were waiting for such solicitation. Their procrastination has figured as an asset only in good intention. The liberal subscriptions made by a comparatively small number of our members shows the great interest in and need felt for this home, and I feel certain, assures the ultimate success of this project. Our permanent fund is slowly increasing, our membership growing, the unanimity of the profession greater and but a short time is needed to see the fulfilment of this project. The Academy, I feel, will not assume the dignity and standing in the community which it deserves until the hopes of the committee have been realised. The thanks of the organisation are certainly due this committee who have labored so long and earnestly in its behalf.

The collation committee has provided five luncheons during the year, which have added to the social side of our meetings and enabled the members to become better acquainted.

The commissions on milk supply, on inspection of schools and school children and on the reporting of tuberculosis have undertaken problems for the advancement of sanitary medicine which will improve our city. The Academy should not be ungrateful to those who have served it in these various capacities.

By action of the Academy last year it was deemed wise that we should become an incorporated body so that, should occasion arise, real estate or other property might be held in the name of the organisation. After due legal process, articles of incorporation were granted and we became a duly incorporated body November 10, 1908, the official title being, Buffalo Academy of Medicine.

The financial affairs of the organisation are in a prosperous condition. The permanent fund has been increased by several hundred dollars through the accession of new members. In other years it has been found possible to add to the permanent fund from excess of membership dues over current expenses. We regret that this has not been possible during this year. This is explained on the ground that there have been several extraordinary items of expense. The cost of incorporation and of an option on the High Street site were expensive. The addition of eighty-five new members, whose initiation fees have gone entire to the permanent fund, has by addition to the printing and postage proven a drain upon the current fund The increased attendance at the meetings has incurred greater expense for collations when they have been served. I desire personally to thank both the secretary and the treasurer who have been most attentive and energetic in the prosecution of their duties.

The Academy has during the year accepted invitations and been entertained by the Clinical Club and by the Erie County Pharmaceutical Society. At these meetings scientific papers were read and discussed, the social side in medicine has been cultivated and all who attended these meetings were benefited and given a better opinion of the activities of our fellow organisations. The meeting with the latter body has certainly been productive in sentiment against proprietary remedies.

The year has been one of particular pleasure to me. The utmost consideration has been shown my many failings. The loyalty and coöperation of members at every hand has been most gratifying. Men already overworked have undertaken willingly additional tasks to further the Academy's interests. The year has been free from all contentions. Harmony has been universal. It has been a busy year and many problems have arisen. They have been met by our members in a broad-minded and liberal manner.

Our membership 339, and an increase of 85 members for the year, marks the greatest prosperity in the history of the Academy. A larger proportion of our members than ever before have become regular weekly attendants of our meetings. They have made their presence secondary only to the demands of their professional duties. This desire to assemble regularly, listen to and discuss theses on live medical topics, shows a healthy condition and progressive spirit in the profession of our city. It shows a general desire to keep abreast of the times, to know and to sift all that is good in scientific research and modern medicine. It shows a general recognition of the advancement of medical science. It shows a general tendency to throw off the petty jealousies for which our profession has been criticised for ages.

In closing allow me to bespeak for my successor the same generous treatment which has been accorded me—the same loyalty of members and the same earnest, energetic coöperation ot fellow officers. With this, our growth will be ever more rapid than it has been in the past, the sphere of usefulness of the Academy will be manifoldly increased, and the influence of our organisation will become unlimited in shaping the medical and sanitary affairs of Buffalo.

234 E. Utica Street.

